# Heat Stress Targeting Individual Organs Reveals the Central Role of Roots and Crowns in Rice Stress Responses

**DOI:** 10.3389/fpls.2021.799249

**Published:** 2022-01-17

**Authors:** Sylva Prerostova, Jana Jarosova, Petre I. Dobrev, Lucia Hluskova, Vaclav Motyka, Roberta Filepova, Vojtech Knirsch, Alena Gaudinova, Joseph Kieber, Radomira Vankova

**Affiliations:** ^1^Laboratory of Hormonal Regulations in Plants, Institute of Experimental Botany, Czech Academy of Sciences, Prague, Czechia; ^2^Department of Biology, University of North Carolina at Chapel Hill, Chapel Hill, NC, United States

**Keywords:** acclimation, antioxidant enzymes, cytokinin oxidase/dehydrogenase (CKX), gene expression, heat shock, jasmonoyl-isoleucine, *Oryza sativa* (L.), phytohormones

## Abstract

Inter-organ communication and the heat stress (HS; 45°C, 6 h) responses of organs exposed and not directly exposed to HS were evaluated in rice (*Oryza sativa*) by comparing the impact of HS applied either to whole plants, or only to shoots or roots. Whole-plant HS reduced photosynthetic activity (F_*v*_/F_*m*_ and QY__*Lss*_), but this effect was alleviated by prior acclimation (37°C, 2 h). Dynamics of *HSFA2d*, *HSP90.2*, *HSP90.3*, and *SIG5* expression revealed high protection of crowns and roots. Additionally, *HSP26.2* was strongly expressed in leaves. Whole-plant HS increased levels of jasmonic acid (JA) and cytokinin *cis*-zeatin in leaves, while up-regulating auxin indole-3-acetic acid and down-regulating *trans*-zeatin in leaves and crowns. Ascorbate peroxidase activity and expression of alternative oxidases (*AOX*) increased in leaves and crowns. HS targeted to leaves elevated levels of JA in roots, *cis*-zeatin in crowns, and ascorbate peroxidase activity in crowns and roots. HS targeted to roots increased levels of abscisic acid and auxin in leaves and crowns, *cis*-zeatin in leaves, and JA in crowns, while reducing *trans*-zeatin levels. The weaker protection of leaves reflects the growth strategy of rice. HS treatment of individual organs induced changes in phytohormone levels and antioxidant enzyme activity in non-exposed organs, in order to enhance plant stress tolerance.

## Introduction

Plants are frequently exposed to temperatures above their growth optimum as a result of seasonal and daily temperature fluctuations. Heat stress (HS) is an acute adverse condition that can cause protein denaturation and increased production of reactive oxygen species (ROS), negatively influencing photosynthetic capacity and causing metabolic imbalances (Cortleven et al., 2019). The severity of HS depends on its intensity and duration as well as the rate of temperature increase (Wahid et al., 2007). The early response to a sudden rise in temperature is initiated within 3–5 min and involves the activation of heat shock factors that induce production of heat shock proteins (HSPs; Sachs and Ho, 1986). Many HSPs function as chaperones, which facilitate proper folding of enzymes that might otherwise aggregate or be misfolded under HS conditions. HSP90 also affects the signal transduction of various phytohormones; for example, it stabilises the auxin receptor TIR1 by direct interaction (di Donato and Geisler, 2019) and is necessary for maintenance of the dephosphorylated (active) state of the brassinosteroid-induced transcription factor BES1 (Shigeta et al., 2015).

Phytohormones play central roles in the interaction of plants with their environment. Studies on the involvement of phytohormones in HS responses have focused particularly on the stress hormones abscisic acid (ABA) and ethylene, which have been reported to increase HS tolerance in plants (Larkindale and Knight, 2002). ABA induces substantial transcriptomic changes, stimulating the production of protective compounds including heat shock factors (HSFs) and HSPs (Chauhan et al., 2011), and also regulates stomatal aperture, which is particularly important during the early stages of the HS response. Pre-treatment with salicylic acid (SA) has repeatedly been found to improve heat tolerance in SA-deficient plants (Clarke et al., 2004; Larkindale and Huang, 2004). HS also increases levels of 12-oxo-phytodienoic acid, a precursor of jasmonic acid (JA). In a positive feedback loop, JA promotes the accumulation of several HSPs (Muench et al., 2016). JA signal transduction is mediated by JA conjugate with isoleucine, JA-Ile, which binds to the receptor allowing degradation of repressor proteins JAZ (Wasternack and Song, 2017).

Another phytohormone important in HS responses is auxin: HS stimulates the *YUCCA* auxin biosynthetic genes (Blakeslee et al., 2019). Their up-regulation is enhanced by phytochrome-interacting factor PIF4, which plays a role in both shade and HS responses (Sun et al., 2012). Further, treatment with exogenous auxin alleviates the negative effects of stress (Sharma et al., 2018). Brassinosteroids also promoted HS tolerance, by increasing ROS scavenging capacity (Bajguz and Hayat, 2009), and enhanced thermotolerance via the activity of the BZR1 transcription factor (Yin et al., 2018). Crosstalk between brassinosteroids and ABA is mediated by hydrogen peroxide, which serves as a signalling molecule (Zhou et al., 2014).

Finally, positive effects of cytokinins (CKs) have been observed during long-term HS treatment (e.g., Rivero et al., 2007; Xu et al., 2009). CKs exhibit multiple functions in the HS responses, including stabilisation of the photosynthetic machinery and up-regulating the antioxidant system (Cortleven et al., 2019). In our previous study on early HS response, we demonstrated transient increase in the levels of active CKs in leaves within 30–45 min after HS initiation. This was accompanied by an increase in stomatal conductance that enhanced transpiration, which is the main leaf cooling mechanism. This reaction allows plants to maintain the leaf temperature below that of the environment until other defence mechanisms can be activated, starting with up-regulation of heat shock transcription factors (Macková et al., 2013; Dobrá et al., 2015). The role of CKs was confirmed by studying transformants with inducible CK biosynthesis (dexamethasone-inducible *ipt* expression; Skalák et al., 2016) and by treatment with INCYDE, an inhibitor of the CK degradation enzyme cytokinin oxidase/dehydrogenase (CKX) (Prerostova et al., 2020). Nevertheless, the role of active CKs in HS responses can be complicated by the fact that they differ in their affinity to specific receptors as well as in localisation of their biosynthesis (roots/leaves, plastid/cytoplasm). trans-Zeatin (tZ) is the predominantly root-born CK, most physiologically active in stimulation of cell division. cis-Zeatin (cZ) has much lower growth stimulatory activity than tZ, which is important in the stress conditions associated with growth arrest. However, cZ exhibits other CK functions, e.g., stimulation of antioxidant system or stabilisation of photosynthetic apparatus. Isopentenyladenine is synthesised in aboveground tissues, being slightly less active than tZ (Kieber and Schaller, 2014).

Individual plant organs differ in their physiological functions and sensitivity as well as responses to stresses (Bellstaedt et al., 2019; Vives-Peris et al., 2020). Leaves are the organs that regulate transpiration by adjusting the stomatal aperture, which is important in the early phase of the HS response. They are also sensitive to HS because the photosynthetic apparatus is highly vulnerable to thermal damage (Sun and Guo, 2016). High temperature limits photosynthetic CO_2_ fixation, reducing electron transport beyond plastochinon Q_*A*_, which results in a strong accumulation of H_2_O_2_ in leaves (Sun et al., 2016). Yoshida et al. (2007) suggested that the alternative oxidase (AOX), an important mitochondrial component of the cell stress responses (Vanlerberghe and McIntosh, 1997), acts as a sink to absorb the excess of reducing equivalents in photosynthetic chain. Zhang et al. (2012) demonstrated that the AOX pathway functions in protecting photosystem II (PSII) from photoinhibition by draining electrons away from the electron acceptors at the acceptor side of PSI to sustain linear electron transport, which in turn causes the release of more protons into the lumen and accelerates the consequential induction of non-photochemical quenching (NPQ). The main functions of the roots are the transport of water and nutrients, producing various metabolites (including protective ones), and anchoring the plant in the soil (although the latter function is not relevant in Araponics-type hydroponic systems, used in this work). Unique stress responses are observed in both the root apical meristem and the shoot apical meristem or crown, respectively. Crown is a specific meristematic tissue of cereals in which stems, leaves and adventitious roots are originated. Meristematic tissues are crucial for plant development and are therefore preferentially protected to maximise the likelihood of the plant’s survival under unfavourable conditions. For example, preferential protection of the apices enables the halophyte *Thelungiella halophila* to survive salt stress better than the glycophyte Arabidopsis (Prerostova et al., 2017).

Stress can have different impacts on individual plant organs. Additionally, different organs may not be exposed to stresses by the same degree, particularly during the initial stress phase. For example, harsh frosty winds may have stronger effects on leaves than on roots buried in soil whose temperature remains above 0°C. Similarly, leaves may be exposed to more severe HS than roots on a sunny spring day. Plants have adjusted to these situations by developing rapid inter-organ communication to combat stress conditions. While the stress responses of organs that are directly exposed to stress have been well characterised, the impact on organs that are less exposed or non-exposed to stresses has received less attention (e.g., geothermal *Agrostis*; Rachmilevitch et al., 2006). We have previously demonstrated the occurrence of CK/ABA crosstalk in Arabidopsis plants subjected to targeted HS in the early phase of the response (Dobrá et al., 2015; Skalák et al., 2016). The application of mild HS to roots was reported to have beneficial effect on shoot growth (e.g., Bumgarner et al., 2011).

The aim of this study was to elucidate the inter-organ communication and complex hormonal crosstalk in HS-exposed and non-exposed organs of rice (*Oryza sativa*). HS was applied to shoots, roots, or the whole plant, either directly or after previous acclimation to higher temperature in order to test the influence of acclimation to inter-organ communication. The plant responses were characterised at the level of phytohormones (ABA, JA, SA, auxin, CK, and their metabolites), transcription of stress- and hormone-related genes, photosynthetic parameters, the activity of the CK degradation enzyme CKX and selected antioxidant enzymes.

## Materials and Methods

### Plant Material and Experimental Conditions

*Oryza sativa* L. ssp. japonica (Kitaake) seeds were soaked in distilled water for 1 day and then cultivated for 2 weeks in an Araponics hydroponic system (1.7 L vessel, 18 plants per vessel; 1/4 Hoagland solution) in a Sanyo MLR-350H climate chamber (Sanyo Electric Co.) providing a 14/10 h light/dark photoperiod with an optimal light intensity of 150 μmol m^–2^ s^–1^, a temperature of 25/20°C, and ca. 70% relative humidity. The experiment began on the 15th day after sowing, 2 h after dawn, at the two-leaf stage of development. The medium was changed 4 h after dawn (corresponding to the beginning of the HS treatment). Acclimation (A) was achieved by incubating the plants at 37°C for 2 h. HS (45°C, 6 h, 4 h after dawn) was then applied either to the whole plant (medium pre-heated to 45°C and chamber adjusted to 45°C, HS-WP), to the leaves (a medium temperature maintained at 25°C in insulated vessels, chamber adjusted to 45°C, HS-L; Supplementary Figure 1), or to the roots (medium pre-heated to 45°C and maintained at this temperature in the insulated vessels, chamber adjusted to 25°C, HS-R). Temperature and duration of HS were selected in preliminary experiments, so that HS-WP resulted in permanent growth arrest and lesions on the first leaf, but plants were still able to recover. Acclimated plants were significantly less affected by HS (stopped growth, minimal lesions on leaves) and exhibited faster recovery. Potential effects of circadian rhythms were minimised by comparing stress-treated samples to controls at the same time point (corresponding to the end of HS). The experiment was repeated three times.

For each sample, the crowns, roots, and middle sections of the first leaves (without the bases and tips) from three plants were collected, frozen in liquid nitrogen, and stored at −80°C. Antioxidant enzymes activity, phytohormone levels, gene expression by RT-qPCR, and CKX activity were determined.

### Chlorophyll Fluorescence Measurement

Nine plants (three from each experiment) were dark-adapted for 15 min under the conditions of their treatment. First leaves were subjected to fluorescence measurements with Handy FluorCam FC 1,000-H (Photon Systems Instruments) in Pulse-Amplitude-Modulated Mode. Chlorophyll fluorescence was measured during the dark adaptation phase (duration 5 s), which was followed by a saturating pulse (2,000 μmol m^–2^ s^–1^; 800 ms), dark relaxation (60 s), and an actinic light period (200 μmol m^–2^ s^–1^, duration 400 s) with 9 saturating flashes. The maximum photosystem II (PSII) quantum yield in the dark-adapted state (F_*v*_/F_*m*_) and steady-state PSII quantum yield in the light (QY__*Lss*_) were calculated according to Genty et al. (1989). Steady-state non-photochemical quenching in the light (NPQ__*Lss*_) was measured according to Horton and Ruban (1992).

### Phytohormone Analysis

Phytohormone analysis was done according to Prerostova et al. (2020). Briefly, samples (ca. 10 mg FW; two biological samples per experiment, giving 6 repetitions in total) were homogenised and extracted with cold (-20°C) methanol/water/formic acid (15/4/1 v/v/v). The following isotope-labelled standards were added at 10 pmol per sample: ^13^C_6_-IAA (Cambridge Isotope Laboratories); ^2^H_4_-SA (Sigma-Aldrich); ^2^H_3_-PA, ^2^H_3_-DPA (NRC-PBI); ^2^H_6_-ABA, ^2^H_5_-JA, ^2^H_5_-tZ, ^2^H_5_-tZR, ^2^H_5_-tZRMP, ^2^H_5_-tZ7G, ^2^H_5_-tZ9G, ^2^H_5_-tZOG, ^2^H_5_-tZROG, ^15^N_4_-cZ, ^2^H_3_-DZ, ^2^H_3_-DZR, ^2^H_3_-DZ9G, ^2^H_3_-DZRMP, ^2^H_7_-DZOG, ^2^H_6_-iP, ^2^H_6_-iPR, ^2^H_6_-iP7G, ^2^H_6_-iP9G, ^2^H_6_-iPRMP, ^2^H_2_-GA_19_, (^2^H_5_)(^15^N_1_)-IAA-Asp and (^2^H_5_)(^15^N_1_)-IAA-Glu (Olchemim). The extracts were centrifuged for 20 min at 4°C and 17,000 g and concentrated using an Alpha RVC vacuum centrifuge (Christ; 40°C, 15 mbar, 1.5 h). Phytohormones were separated using a reverse-phase–cation exchange SPE column (Oasis-MCX, Waters), yielding an acid fraction eluted with methanol and a basic fraction eluted with 0.35 M NH_4_OH in 60% methanol. The fractions were dried in the vacuum centrifuge and resuspended in 30 μL acetonitrile (15%) in the case of the acid fraction and in 5% methanol in the case of the basic fraction. Hormone contents were analyzed using HPLC (Ultimate 3,000, Dionex) coupled to 3200 Q TRAP hybrid triple quadrupole/linear ion trap mass spectrometer (Applied Biosystems). Quantification was achieved using the isotope dilution method with multilevel calibration curves (*R*^2^ > 0.99) and the Analyst 1.5 software package (Applied Biosystems).

### Gene Expression Quantification

Samples (ca. 100 mg FW; two biological samples per experiment, giving 6 replicates in total) were homogenised with zirconia balls in a cooled MM301 ball mill (Retsch) for 150 s at 25 Hz. RNA was isolated using RNeasy Plant Mini Kit (Qiagen) and treated with DNase I recombinant (Roche Applied Science) in accordance with the manufacturer’s instructions. Total mRNA was translated to cDNA using M-MLV Reverse Transcriptase (RNase-H Minus, Point Mutant, Promega), oligo dT primers, and Protector RNase Inhibitor (Roche Applied Science). The cDNA (diluted 10-fold) was mixed with 5 μL GoTaq qPCR Master Mix (Promega) and specific primers (Supplementary Table 1; Jain et al., 2006; Chauhan et al., 2011; Li et al., 2013; Xu et al., 2015; Zhang H. et al., 2016; Ghosh et al., 2018; Kumar et al., 2018) to a final volume of 10 μL. Target sequences were amplified by PCR with cycles of 10 s at 95°C for primer denaturation and 30 s at 60°C for annealing and elongation using Light Cycler 480 (Roche Applied Science). The eukaryotic translation initiation factor *eIF5A* and E2 ubiquitin-conjugating enzyme *UBC-E2* were selected as reference genes based on expression stability across the samples, which was evaluated using RefFinder (Xie et al., 2012) and the Genevestigator database (Hruz et al., 2008). Relative RNA contents were calculated according to Pfaffl (2001) (Supplementary Table 2).

### Measurement of Antioxidant Enzyme Activities

About 300 mg FW of first leaves, crowns and roots were used for isolation of antioxidant enzymes. Three replicates were collected (one per independent experiment). Samples were homogenised in 0.1 M Tris-HCl buffer containing 3 mM MgCl_2_, 1 mM EDTA, and 5 mM ascorbic acid in a 1:5 FW ratio. The homogenate was centrifuged at 14,000 g, 4°C for 20 min, and the supernatant was collected. The protein concentration was determined using Protein Assay Dye Reagent Concentrate (Bio-Rad) and measured on a spectrophotometer at 595 nm (Bradford, 1976) using bovine serum albumin as a standard.

Catalase (CAT, EC 1.11.1.6) activity was measured by monitoring the decrease in H_2_O_2_ concentration by spectrophotometry at 240 nm in a quartz cuvette (Adam et al., 1995). Ascorbate peroxidase (APX, EC 1.11.1.11) activity was measured by monitoring the decrease in ascorbate concentration by spectrophotometry at 290 nm in a quartz cuvette (Nakano and Asada, 1987). Superoxide dismutase (SOD, EC 1.15.1.1) activity was determined according to Beauchamp and Fridovich (1971) using nitrotetrazolium blue chloride (NBT) as a marker of superoxide radicals. Activity was measured by spectrophotometry at 560 nm and calculated according to Zhang C. et al. (2016).

### Cytokinin Oxidase/Dehydrogenase Activity

CKX (EC 1.5.99.12) was extracted from frozen samples (three independent biological samples) and purified according to Motyka and Kaminek (1992, 1994) and Motyka et al. (2003). Enzyme activity was determined by *in vitro* radioisotope assays based on the conversion of tritium-labelled *N*^6^-(Δ^2^-isopentenyl) adenine ([^3^H_2_]iP) (prepared by the Isotope Laboratory, Institute of Experimental Botany of the Czech Academy of Sciences, Prague, Czech Republic) to adenine. The assay mixture (50 μL) comprised 100 mM TAPS-NaOH buffer containing 75 μM 2,6-dichloroindophenol (pH 8.5), 2 μM substrate [^3^H_2_]iP, and enzyme preparation equivalent to 4 (roots) or 20 mg (leaves and crowns) tissue FW. After incubation (1 h at 37°C), the reaction was terminated by adding 10 μL Na_4_EDTA (200 mM) and 120 μL 95% (v/v) ethanol. The substrate was separated from the product of the enzyme reaction by HPLC, as described in Gaudinova et al. (2005).

### Statistical Analyses

Differences in measured variables related to the treatments and plant organs were analysed by two-way ANOVA with Tukey’s *post hoc* test (*p* < 0.05) using Prism 8 (GraphPad). Numbers of independent biological replicates in specific analyses are specified above. Principal component analysis (PCA) was calculated from active hormones (ABA, IAA, SA, JA, JA-Ile, tZ, and cZ) using Prism 8. Data for PCA were standardised in order to adjust phytohormones of different scale.

## Results

### Photosynthetic Parameters

**FIGURE 1:**
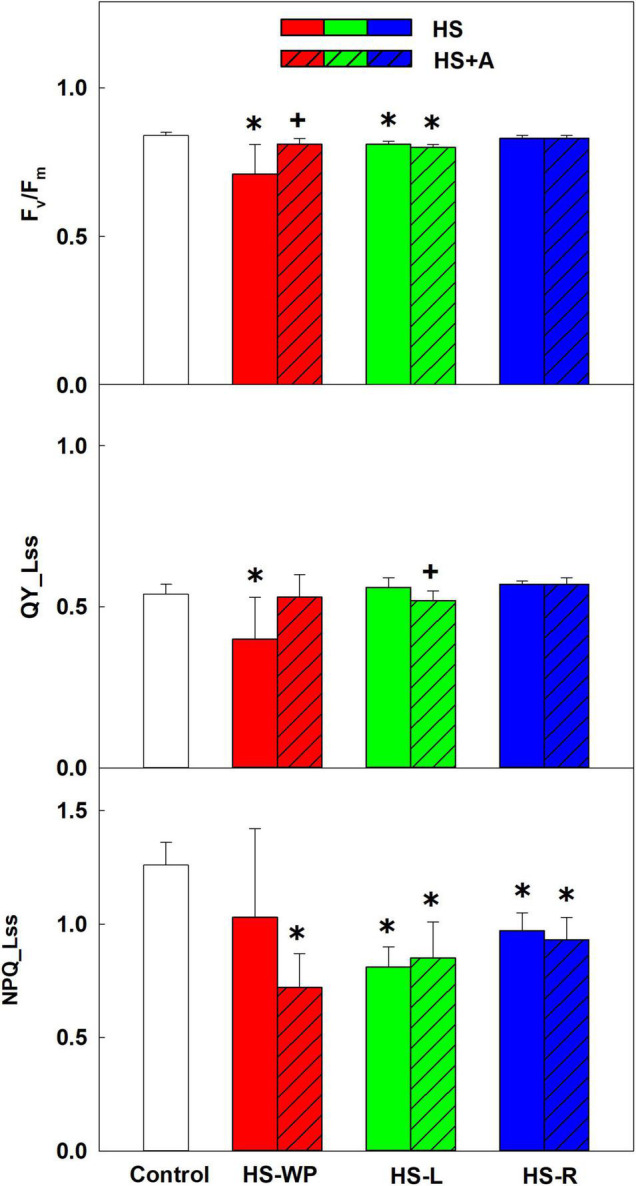
Photosynthetic parameters: the maximum quantum yield of PSII in the dark-adapted state (F_*v*_/F_*m*_), steady-state quantum yield (QY__*Lss*_) and non-photochemical quenching (NPQ__*Lss*_) of PSII. Rice seedlings were exposed to heat stress directly (HS) or after acclimation (HS + A). Heat stress was applied to whole plants (HS-WP), to leaves (HS-L), or to roots (HS-R). The first leaf was measured. Data are means ± SD. One-way ANOVA with Tukey’s *post hoc* test (*p* < 0.05, *n* = 9) was used to compare controls to the HS treatments. Significant differences compared to control plants are indicated by asterisks (*) while crosses (+) indicate significant differences between HS and HS + A treatments.

The inter-organ crosstalk after applying HS to leaves (HS-L) or roots (HS-R) was compared to that observed after whole-plant HS (HS-WP). Moreover, the impact of previous acclimation by high temperature (37°C for 2 h) to organ-specific responses was investigated. The photosynthetic parameters were determined in the first leaves. The negative impact of HS-WP on maximal quantum yield (F_*v*_/F_*m*_) was much stronger than those of the other stresses (Figure 1). However, acclimation almost completely abolished this negative effect. Conversely, HS-L and HS-R had only slight effects on F_*v*_/F_*m*_. In HS-R, acclimation had no significant effect. Similar results were obtained for the steady state quantum yield (QY__*Lss*_), which decreased markedly after HS-WP treatment, but prior acclimation allowed it to remain comparable to that seen in control plants. The level of non-photochemical quenching (NPQ__*Lss*_) was lower in all HS treatments than under control conditions, being the lowest in the acclimated plants treated by HS-WP.

### Plant Hormones

**FIGURE 2:**
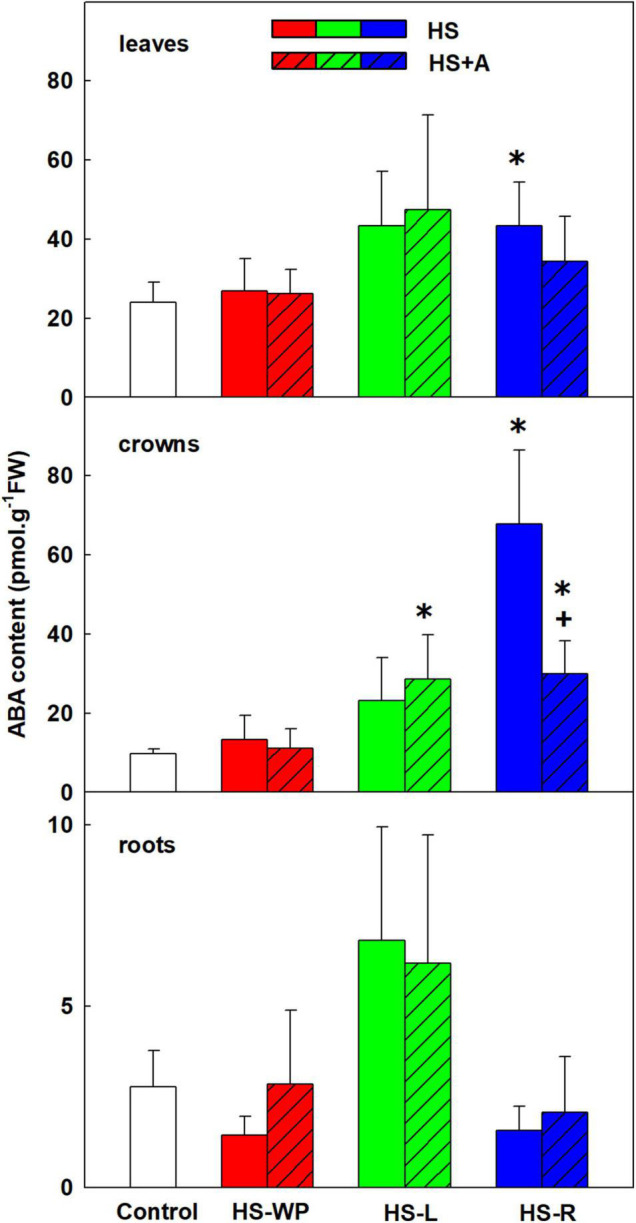
The content of abscisic acid (ABA) in leaves, crowns, and roots of rice seedlings exposed to heat stress directly (HS) or after acclimation (HS + A). Heat stress was applied to whole plants (HS-WP), to leaves (HS-L), or to roots (HS-R). Data are means ± SD. One-way ANOVA with Tukey’s *post hoc* test (*p* < 0.05, *n* = 6) was used to compare controls to the HS treatments. Significant differences compared to control plants are indicated by asterisks (*) while crosses (+) indicate significant differences between HS and HS + A treatments.

ABA plays a crucial role in abiotic stress responses. However, its levels after 6 h of direct HS-WP treatment did not differ significantly from those in control plants (Figure 2). Conversely, the HS-L treatment increased ABA levels in all tested organs, although this effect was significant only in the crowns. Simultaneously, ABA catabolite dihydrophaseic acid (DPA) was up-regulated after HS-WP in leaves, being at control level after HS-L (Supplementary Table 3). The HS-R treatment had a similar positive impact on ABA as HS-L in leaves, causing the strongest increase in ABA levels in crowns, especially after direct HS treatment. The levels of ABA did not significantly differ from controls in roots, while both phaseic acid and DPA were very low.

**FIGURE 3:**
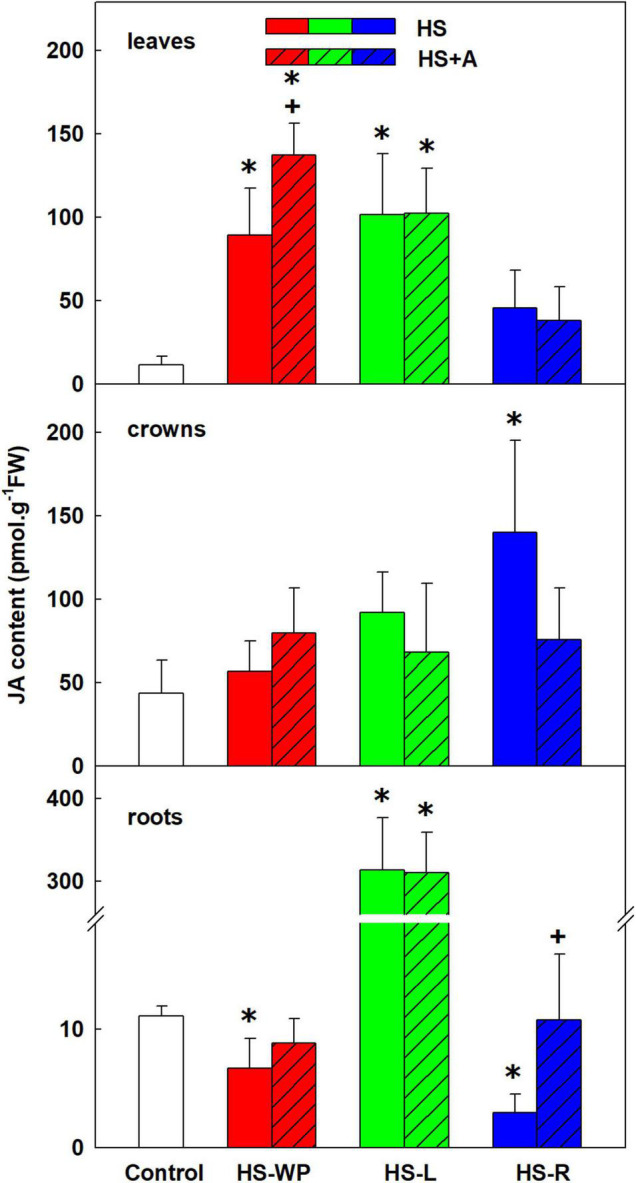
The content of jasmonic acid (JA) in leaves, crowns and roots of rice seedlings exposed to heat stress directly (HS) or after acclimation (HS + A). Heat stress was applied to whole plants (HS-WP), to leaves (HS-L) or roots (HS-R). Data are means ± SD. One-way ANOVA with Tukey’s *post hoc* test (*p* < 0.05, *n* = 6) was used to compare controls to the HS treatments. Significant differences compared to control plants are indicated by asterisks (*) while crosses (+) indicate significant differences between HS and HS + A treatments.

All of the heat treatments increased JA levels in leaves, but the effect was most significant for HS-WP, in which case acclimation had a distinct strengthening effect (Figure 3). In crowns, the strongest increase in JA levels was induced by HS-R; HS-WP and HS-L had no significant effects. HS-L caused very strong JA up-regulation in roots, irrespective of acclimation. In contrast, the other HS treatments caused down-regulation of JA levels after direct stress. The active metabolite involved in JA signalling JA-Ile behaved similarly to JA (Supplementary Figure 2) in all organs except crowns under the HS-WP treatment, where JA-Ile levels increased significantly, especially after acclimation. JA-Ile was significantly down-regulated in HS-exposed roots (i.e., under the HS-WP and HS-R treatments).

**FIGURE 4:**
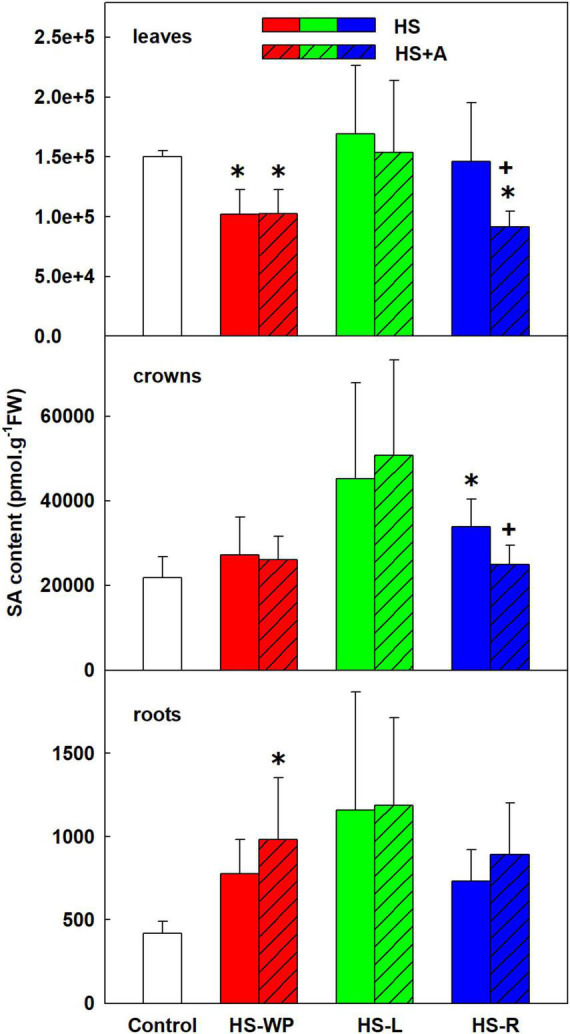
The content of salicylic acid (SA) in leaves, crowns and roots of rice seedlings exposed to heat stress directly (HS) or after acclimation (HS + A). Heat stress was applied to whole plants (HS-WP), to leaves (HS-L), or to roots (HS-R). Data are means ± SD. One-way ANOVA with Tukey’s *post hoc* test (*p* < 0.05, *n* = 6) was used to compare controls to the HS treatments. Significant differences compared to control plants are indicated by asterisks (*) while crosses (+) indicate significant differences between HS and HS + A treatments.

HS-WP had a moderate negative effect on SA levels in leaves (Figure 4). Contrarily, HS-L had no significant impact on SA levels in leaves, and HS-R reduced SA levels only after acclimation. In crowns, SA levels were slightly (but generally non-significantly) increased under the HS-L and direct HS-R treatments. HS treatments tended to elevate SA levels in roots, but a statistically significant up-regulation was only observed in acclimated HS-WP plants. Interestingly, benzoic acid, one of the SA precursors (Widhalm and Dudareva, 2015), did not correlate with SA levels, showing high levels in all tissues after HS-R treatments (Supplementary Table 3). The levels of ethylene precursor aminocyclopropane carboxylic acid (ACC) were elevated after direct HS-WP in leaves and in crowns (irrespective of acclimation). In roots, ACC increased after HS-L and HS-R with preceding acclimation (Supplementary Table 3).

**FIGURE 5:**
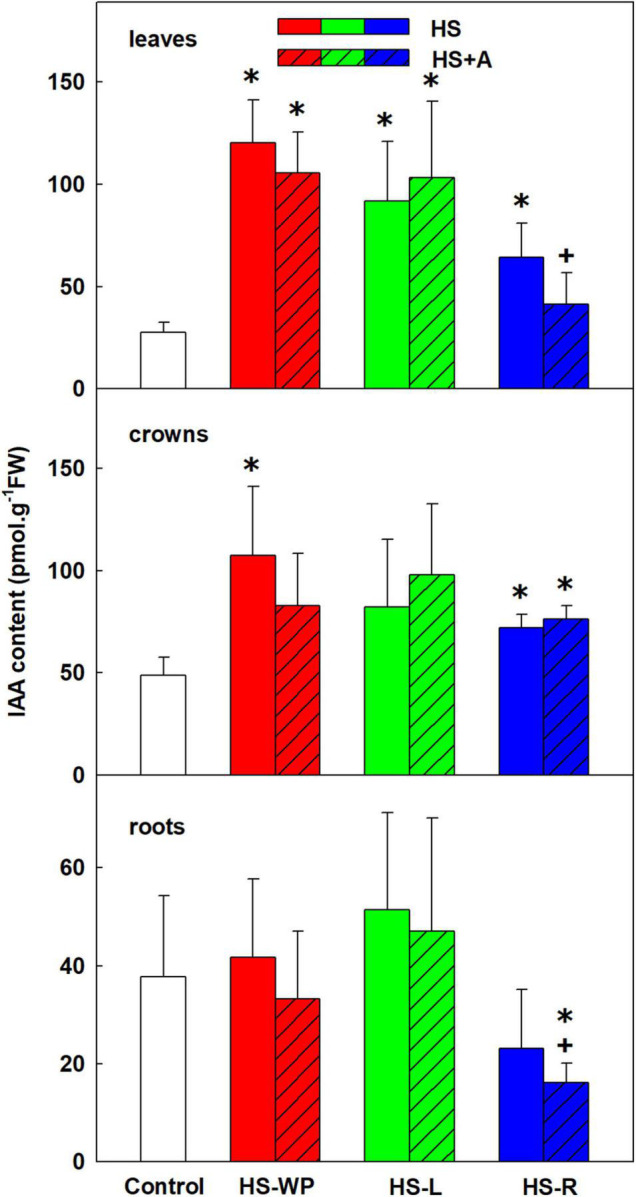
The content of auxin indole-3-acetic acid (IAA) in leaves, crowns and roots of rice seedlings exposed to heat stress directly (HS) or after acclimation (HS + A). Heat stress was applied to whole plants (HS-WP), to leaves (HS-L), or to roots (HS-R). Data are means ± SD. One-way ANOVA with Tukey’s *post hoc* test (*p* < 0.05, *n* = 6) was used to compare controls to the HS treatments. Significant differences compared to control plants are indicated by asterisks (*) while crosses (+) indicate significant differences between HS and HS + A treatments.

All HS treatments, especially HS-WP and HS-L, elevated contents of auxin indole-3-acetic acid (IAA) in leaves; HS-WP and HS-R (and to a lesser extent, HS-L) in crowns (Figure 5). IAA levels in roots were not significantly affected by above-ground HS but were reduced by the HS-R treatment after acclimation. As IAA levels may dynamically change during HS response, the levels of IAA conjugates (IAA-aspartate and IAA-glutamate) and catabolite (oxo-IAA) were followed in order to evaluate IAA regulations (Supplementary Table 3). Both amino acid inactive conjugates were down-regulated by direct HS-WP and HS-R in roots. IAA-aspartate levels were elevated after HS-WP in leaves, and after HS-R in crowns. The catabolite oxo-IAA exhibited relatively minor changes. The levels of low active auxin phenylacetic acid showed similar pattern as IAA (Supplementary Table 3).

**FIGURE 6:**
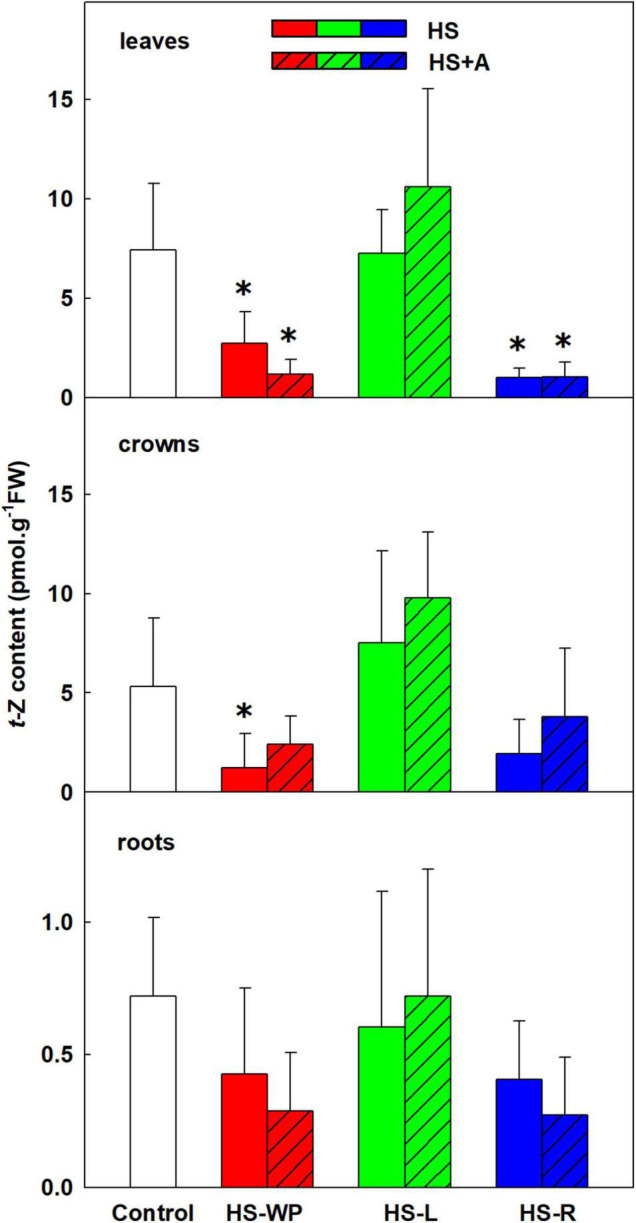
The content of cytokinin *trans-*zeatin (tZ) in leaves, crowns and roots of rice seedlings exposed to heat stress directly (HS) or after acclimation (HS + A). Heat stress was applied to whole plants (HS-WP), to leaves (HS-L), or to roots (HS-R). Data are means ± SD. One-way ANOVA with Tukey’s *post hoc* test (*p* < 0.05, *n* = 6) was used to compare controls to the HS treatments. Significant differences compared to control plants are indicated by asterisks (*).

**FIGURE 7:**
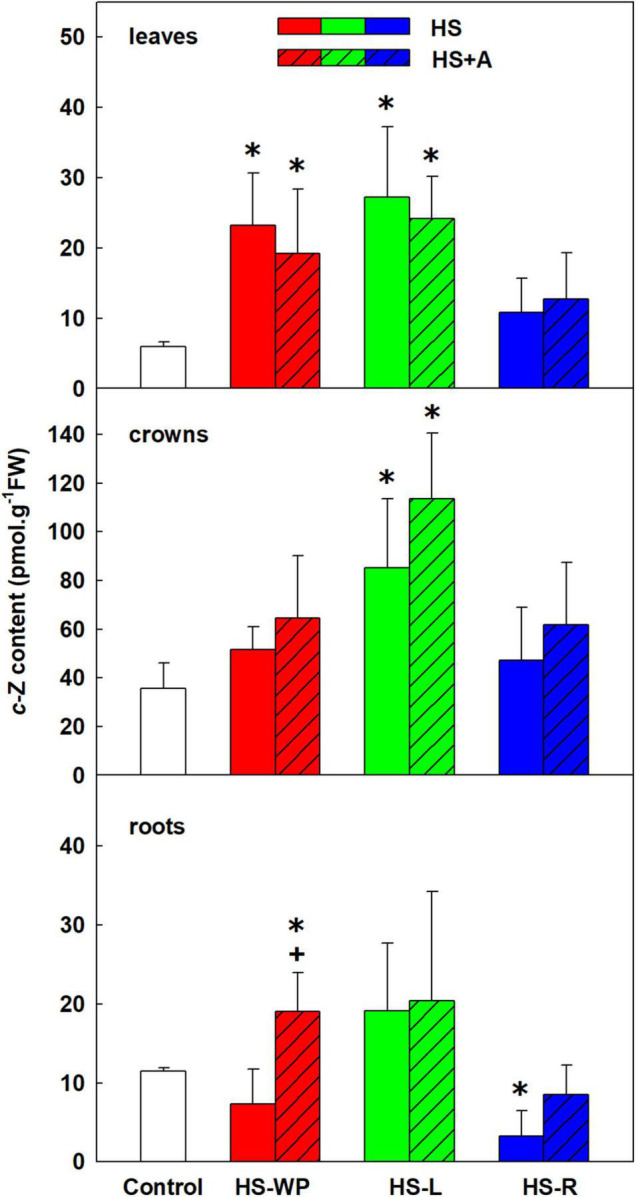
The content of cytokinin *cis-*zeatin (cZ) in leaves, crowns and roots of rice seedlings exposed to heat stress directly (HS) or after acclimation (HS + A). Heat stress was applied to whole plants (HS-WP), to leaves (HS-L), or to roots (HS-R). Data are means ± SD. One-way ANOVA with Tukey’s *post hoc* test (*p* < 0.05, *n* = 6) was used to compare controls to the HS treatments. Significant differences compared to control plants are indicated by asterisks (*) while crosses (+) indicate significant differences between HS and HS + A treatments.

The content of the root-born, highly physiologically active CK tZ was down-regulated in leaves and crowns under the HS-WP and HS-R treatments, although the effect of the HS-R treatment in crowns was not statistically significant (Figure 6). Levels of tZ under the HS-L treatment were similar to those in control plants, with no significant changes detected in the roots. tZ riboside and precursor (tZ riboside phosphate) showed similar down-regulation under HS-WP and HS-R and up-regulation under HS-L treatments in roots and crowns indicating negative HS effect on CK biosynthesis in roots and transport to leaves (Supplementary Table 3). The levels of tZ riboside monophosphate in leaves were around detection limit. HS-L conditions were associated with enhanced tZ deactivation, as indicated by the levels of the deactivation metabolite tZ-9N-glucoside (Supplementary Table 3). HS-WP and HS-L strongly elevated contents of the stress-related CK cZ in leaves (Figure 7), but the strongest increase in cZ levels was seen under HS-L conditions in crowns. The other HS treatments non-significantly increased cZ levels in crowns. Additionally, cZ levels were enhanced slightly in roots under HS-L conditions and also under HS-WP conditions after acclimation. Conversely, direct heat stress (HS-R) reduced cZ levels in roots. Decrease of cZ riboside and the precursor (phosphate) was also found after direct HS-WP and HS-R treatments in roots, while HS-L caused elevation of their levels (Supplementary Table 3). The changes in inactive storage forms [cZ(R) O-glucosides] were insignificant. The levels of leaf-born isopentenyladenine were not significantly affected. Nevertheless, the low levels of its riboside and phosphate indicate its down-regulation by HS-WP and HS-R treatments (Supplementary Table 3). The levels of dihydrozeatin-type CKs were under detection limit.

**FIGURE 8:**
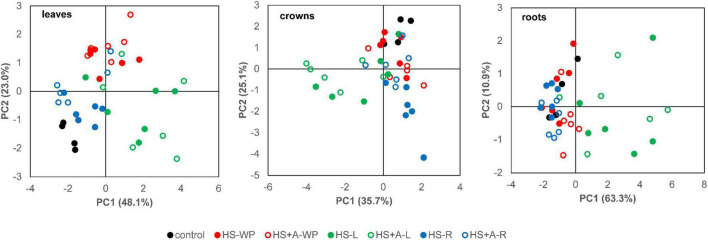
PCA analysis of active hormone levels (ABA, IAA, JA, JA-Ile, SA, tZ, and cZ) in leaves, crowns, and roots of individual samples. Rice seedlings were exposed to heat stress directly (HS) or after acclimation (HS + A). Heat stress was applied to whole plants (WP), leaves (L), or roots (R). Data for PCA were standardised in order to adjust phytohormones of different scale.

PCA of hormonal responses after HS treatments separated HS-WP from controls in leaves, and in the case of the acclimated plants also in roots (Figure 8). Pronounced differences between the controls and HS-L treatments were detected in all organs. HS-R hormonal response differed from that induced by shoot-targeted HS in crowns, especially in directly stressed plants.

### Gene Expression

**FIGURE 9:**
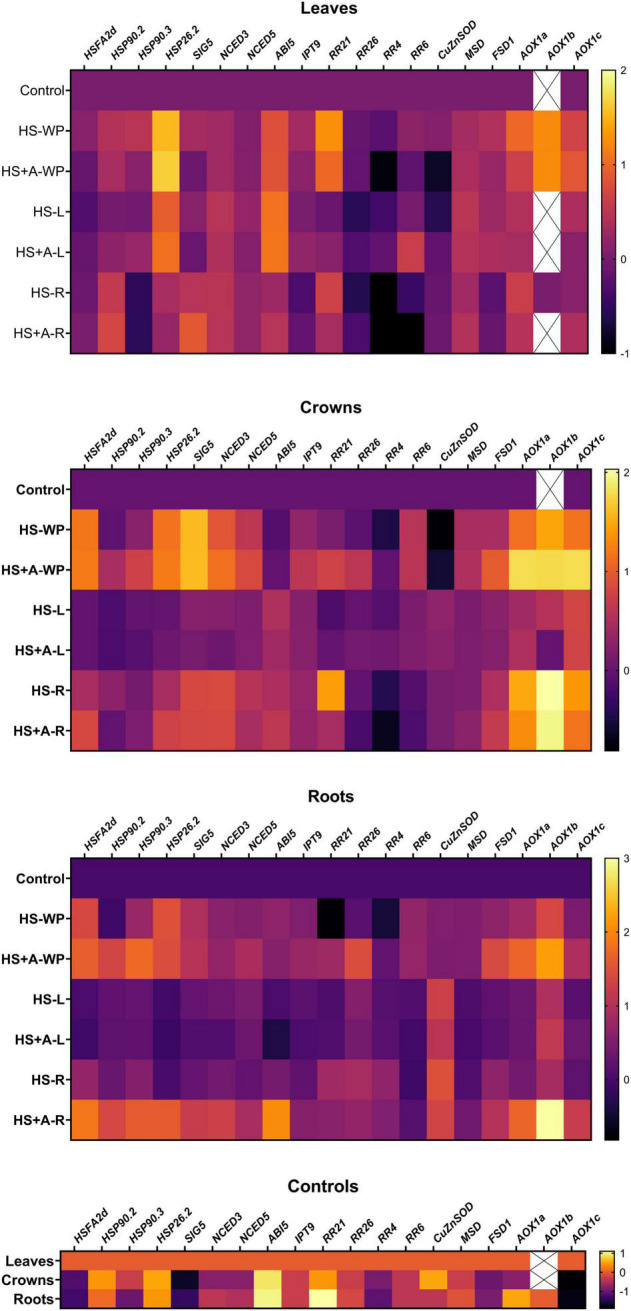
Heatmap of changes in transcript levels of selected stress- and phytohormone-related genes. Gene expression was measured in leaves, crowns, and roots of rice seedlings under control conditions (control) and plants subjected to HS applied to whole plants (HS-WP), whole plants after acclimation (HS + A-WP), to leaves (HS-L), leaves after acclimation (HS + A-L), to roots (HS-R), and roots after acclimation (HS + A-R). Six independent biological samples were evaluated. Data in the heatmap are normalised against control levels separately for each tissue and time-point. The mean values are presented in log_10_ colour scale. Means ± SD and other statistics are shown in Supplementary Table 2. In some experimental groups, the *AOX1b* expression levels of most of the samples were under the detection limits of RT-qPCR, therefore they are not included in the results and marked as X. Heatmap was created using Prism 8 (GraphPad).

Gene transcription heatmaps for the various treatments are presented in Figure 9. The important HS marker gene is heat shock factor *HSFA2d* (Cheng et al., 2015). No significant change in *HSFA2d* expression in leaves was observed after any HS treatment, with or without prior acclimation. However, HS-WP conditions strongly increased its expression in crowns, independently of acclimation. Lesser elevations were observed under HS-R conditions, especially after acclimation. In roots, the HS-WP and HS-R treatments both significantly stimulated *HSFA2d* expression, acclimation having a strong positive effect, especially in the latter case.

The expression of the heat-shock protein *HSP90.2* in leaves increased under HS-R conditions with or without prior acclimation. In roots, acclimation significantly up-regulated *HSP90.2* expression under both HS-R and HS-WP conditions. The same was observed for *HSP90.3* in roots and after HS-WP in crowns. In leaves, expression of the small heat shock protein *HSP26.2* was stimulated strongly by HS-WP and moderately by HS-L, acclimation having a negligible effect. In crowns and roots, HS-WP had a strong positive effect but HS-R caused a strong stimulation only after acclimation, especially in roots.

Sigma factor SIG5 is essential for the expression of the D2 protein in chloroplasts (Nagashima et al., 2004). In roots and especially in crowns, its expression increased strongly under HS-WP conditions, independently of prior acclimation. Acclimation also enhanced *SIG5* expression in all studied organs following HS-R treatment.

No HS treatment significantly changed the expression of the ABA biosynthetic enzymes *NCED3* and *NCED5* in leaves, but their expression in crowns and roots was moderately stimulated under HS-WP conditions, especially after acclimation. The HS-R treatment caused a slightly weaker increase in their expression in crowns and strong elevation in roots after acclimation. Contrarily, expression of the ABA-inducible gene *ABI5* in leaves increased strongly under HS-L conditions and less so under HS-WP conditions. In roots, its expression was up-regulated under HS-R conditions with prior acclimation.

Transcription of the CK biosynthetic gene associated with cZ synthesis *IPT9* increased moderately in crowns and roots under HS-WP conditions, and also in roots under HS-R conditions with acclimation. The CK type-B response regulator *RR21* was up-regulated in leaves after HS-WP and HS-R treatment, and after direct HS-R treatment in crowns. The type-B *RR26* expression was diminished in leaves but elevated in crowns and roots after acclimation followed by HS-WP. Expression of the type-A response regulator *RR4* was strongly suppressed in leaves (and to a lesser degree in crowns) after HS-R. Conversely, HS (especially direct stress) moderately increased *RR4* expression in roots. Expression of type-A *RR6* was only slightly affected; being up-regulated in leaves under HS-L conditions after acclimation and under HS-WP conditions in crowns and roots (with or without prior acclimation).

The HS-R and HS-L treatments both strongly up-regulated expression of cytoplasmic *CuZnSOD*, with the increase being slightly weaker after acclimation. Mitochondrial *MnSOD* (*MSD*) expression was enhanced moderately in crowns (and to a lesser extent in roots) under HS-WP conditions. Transcription of plastid *FeSOD* (*FSD1*) was stimulated by acclimation in crowns and roots by HS-WP treatment and to a lesser extent under HS-R conditions. The expression of the alternative oxidase isoenzymes *AOX1a, AOX1b*, and *AOX1c* was strongly promoted by all HS treatments. All three *AOX* isoenzymes were up-regulated by HS-WP treatment in leaves (independently of acclimation) and by both HS-WP and HS-R in crowns and roots; in the latter organs, the increase was strengthened by prior acclimation.

### Antioxidant Enzyme Activity

**FIGURE 10:**
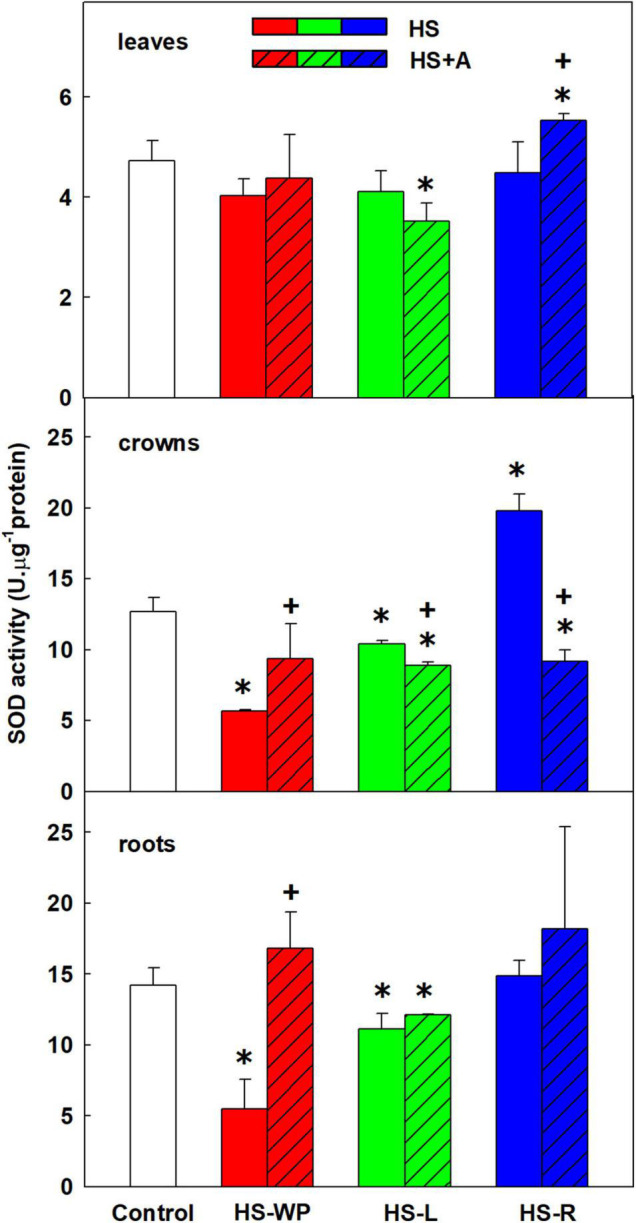
The activity of superoxide dismutase (SOD) in leaves, crowns and roots of rice seedlings exposed to heat stress directly (HS) or after acclimation (HS + A). Heat stress was applied to whole plants (HS-WP), to leaves (HS-L), or to roots (HS-R). Data are means ± SD. One-way ANOVA with Tukey’s *post hoc* test (*p* < 0.05, *n* = 3) was used to compare controls to the HS treatments. Significant differences compared to control plants are indicated by asterisks (*) while crosses (+) indicate significant differences between HS and HS + A treatments.

SOD catalyses conversion of superoxide radicals into molecular oxygen and hydrogen peroxide. The measured SOD activity reflects the activity of all SOD isoenzymes (cytoplasmic, mitochondrial, and plastid). SOD activity increased in leaves after HS-R treatment with prior acclimation (Figure 10). In crowns, SOD activity was significantly up-regulated only by direct HS-R treatment. Both in crowns and roots, SOD activity was strongly suppressed by direct HS-WP treatment, in the latter organ it was moderately reduced under HS-L conditions. Under all HS treatments, acclimation stimulated SOD activity in roots.

**FIGURE 11:**
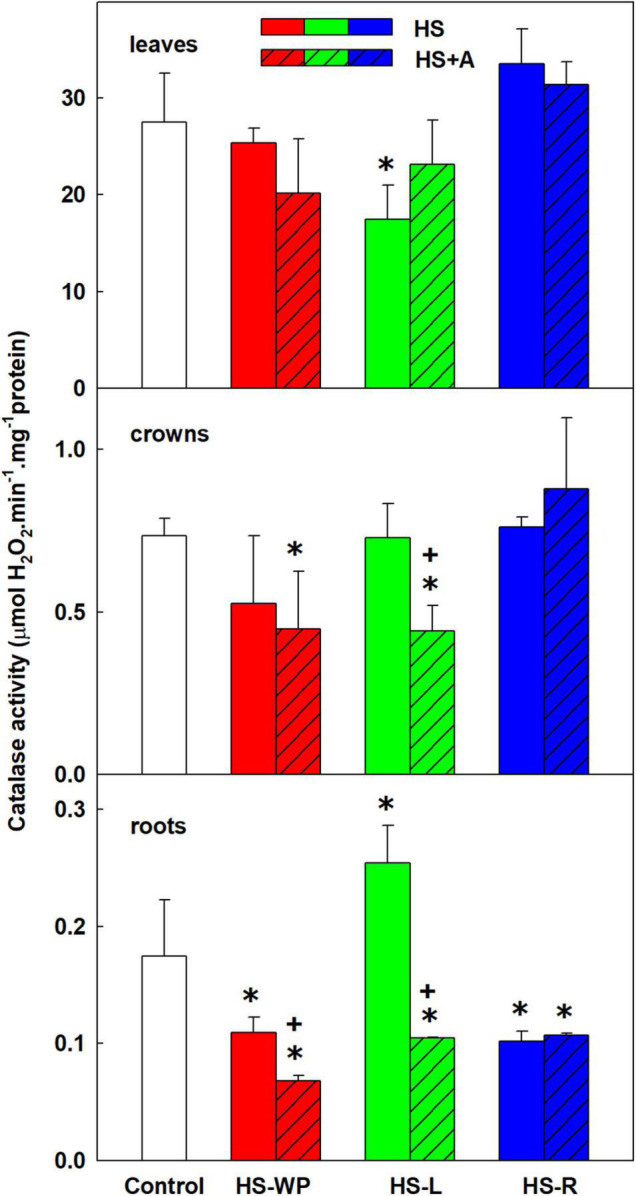
The activity of catalase (CAT) in leaves, crowns and roots of rice seedlings exposed to heat stress directly (HS) or after acclimation (HS + A). Heat stress was applied to whole plants (HS-WP), to leaves (HS-L), or to roots (HS-R). Data are means ± SD. One-way ANOVA with Tukey’s *post hoc* test (*p* < 0.05, *n* = 3) was used to compare controls to the HS treatments. Significant differences compared to control plants are indicated by asterisks (*) while crosses (+) indicate significant differences between HS and HS + A treatments.

CATs are peroxisome protecting enzymes that convert hydrogen peroxide into water and oxygen. Catalase activity was significantly reduced in HS-L stressed leaves (Figure 11). In crowns, CAT activity decreased under HS-WP conditions and after HS-L treatment with prior acclimation. Significant down-regulation of CAT activity was observed in roots under most HS treatments except direct HS-L, which increased CAT activity. These results suggest that catalase activity was enhanced in tissues not exposed to HS.

**FIGURE 12:**
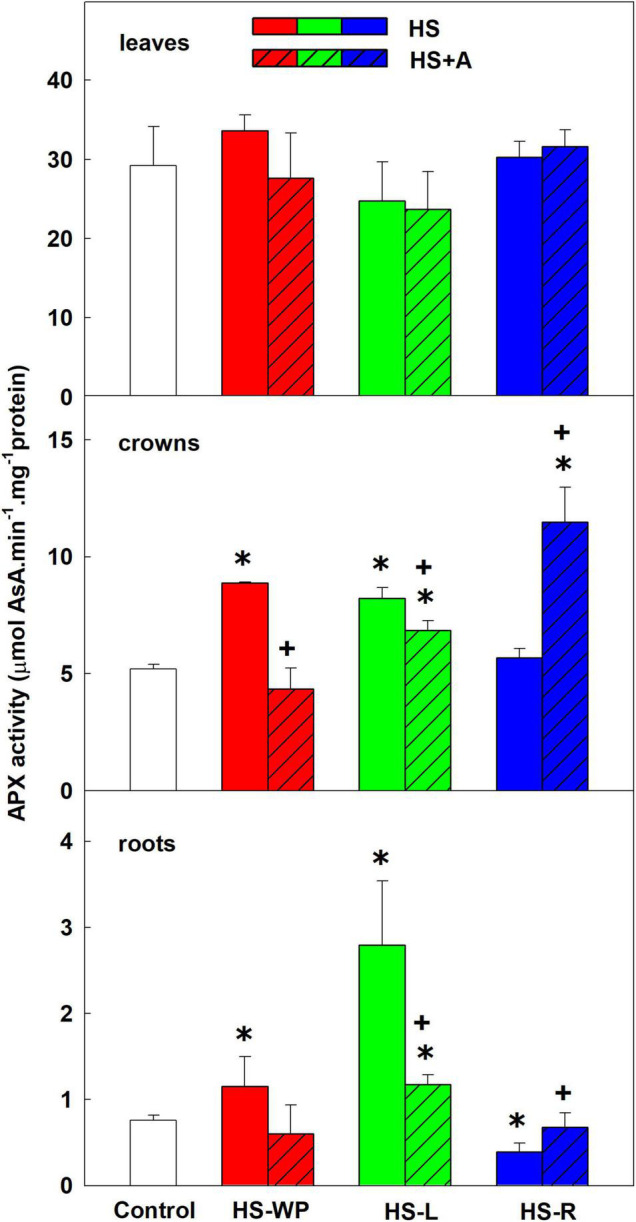
The activity of ascorbate peroxidase (APX) in leaves, crowns and roots of rice seedlings exposed to heat stress directly (HS) or after acclimation (HS + A). Heat stress was applied to whole plants (HS-WP), to leaves (HS-L), or to roots (HS-R). Data are means ± SD. One-way ANOVA with Tukey’s *post hoc* test (*p* < 0.05, *n* = 3) was used to compare controls to the HS treatments. Significant differences compared to control plants are indicated by asterisks (*) while crosses (+) indicate significant differences between HS and HS + A treatments.

APXs also decompose hydrogen peroxide using ascorbate as the reductant. They are localised in chloroplasts, mitochondria, microsomes, and cytoplasm (Shigeoka et al., 2002). APX activity in crowns was increased by direct HS-WP, by HS-L, and especially by HS-R after acclimation (Figure 12). Its activity in roots was also significantly increased by direct HS-L treatment, whereas direct HS-R treatment reduced APX activity.

### Cytokinin Oxidase/Dehydrogenase Activity

**FIGURE 13:**
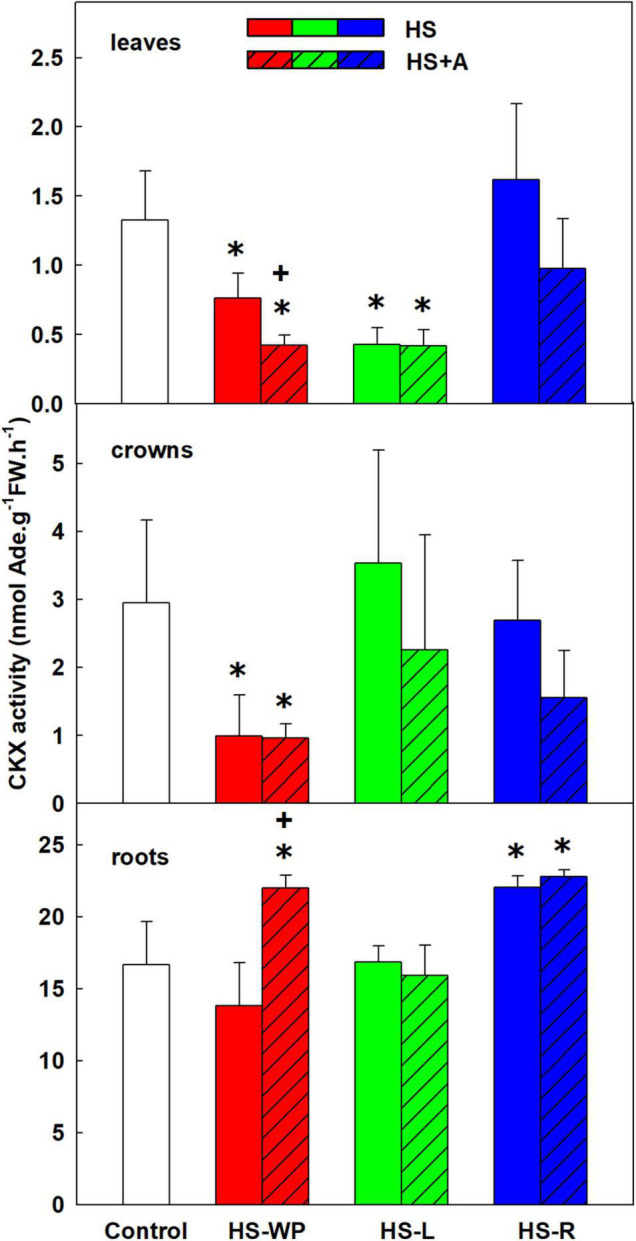
The activity of cytokinin oxidase/dehydrogenase (CKX) in leaves, crowns and roots of rice seedlings exposed to heat stress directly (HS) or after acclimation (HS + A). Heat stress was applied to whole plants (HS-WP), to leaves (HS-L), or to roots (HS-R). Data are means ± SD. One-way ANOVA with Tukey’s *post hoc* test (*p* < 0.05, *n* = 3) was used to compare controls to the HS treatments. Significant differences compared to control plants are indicated by asterisks (*) while crosses (+) indicate significant differences between HS and HS + A treatments.

Besides inactivation by N- or O-glucosylation, CKs can be degraded to adenine and the side chain by CKX enzyme (Motyka and Kaminek, 1992). Applying HS to shoots (HS-WP and HS-L) strongly reduced CKX activity in leaves (Figure 13). In crowns, CKX activity was strongly suppressed by HS-WP treatment irrespective of acclimation. In roots, CKX activity was enhanced by the HS-WP (only with prior acclimation) and HS-R treatments.

## Discussion

### Leaf Stress Responses

Photosynthesis is one of the most HS-sensitive physiological processes. Direct whole-plant stress (HS-WP) had a significant negative effect on photosynthetic parameters in leaves that was accompanied by a moderate increase in the expression of the nuclear-encoded sigma factor *SIG5* (Figures 1, 9). This factor contributes to the repair of damaged PSII reaction centres by stimulating the expression of *psbD*, which encodes the D2 protein (Nagashima et al., 2004). Synthesis of the other stress damageable PSII protein D1 (encoded by *psbA*) is predominantly controlled translationally. *SIG5* expression was reported to be induced by drought, salinity, cold, and high light stress (Kanamaru and Tanaka, 2004). The tendency to preserve the photosynthetic apparatus under HS-WP treatment can be deduced from reduced NPQ__*Lss*_ (Figure 1). Acclimation before HS-WP treatment abolished the negative effects of HS on photosynthetic parameters and reduced the expression of *SIG5* to the control level. Interestingly, HS targeted to leaves (HS-L) had only minor effects on quantum yields. This indicates that maintenance of roots at the control temperature diminishes substantially the stress strength. The HS targeted to roots (HS-R) did not affect quantum yields and moderately up-regulated *SIG5* expression, especially after prior acclimation. It thus seems that HS targeted to roots stimulated defence responses in all plant organs. This is consistent with the conclusions of Vives-Peris et al. (2020), who found that environmental stress conditions trigger various signals in roots that allow plant cells to activate adaptive responses.

A central hormone in abiotic stress responses is ABA. ABA levels in leaves were not increased after 6-h HS-WP and only moderately increased by HS-L, but significant elevation was observed after HS-R (Figure 2). This is consistent with results in Arabidopsis, in which the early phase of the HS response was accompanied with a transient increase in CK and a reduction in ABA levels, leading to a brief increase in stomatal conductance that increased transpiration (Dobrá et al., 2015). This was followed by stomatal closure, presumably to prevent severe water loss, together with down-regulation of CK and increased ABA levels. The dynamics of these changes under HS-WP conditions were considerably faster than under HS-L conditions (Dobrá et al., 2015; Skalák et al., 2016). Additionally, this response was observed only when leaves were exposed to HS (HS-WP, HS-L); under HS-R conditions, ABA levels increased immediately.

The expression of ABA biosynthetic genes (*NCEDs*) in leaves was slightly (but not statistically significantly) increased by the HS-L or HS-R treatments (Figure 9). Despite low ABA levels, the moderate activation of ABA signalling pathway in leaves (evaluated based on the expression of the ABA-inducible gene *ABI5*) was found after HS-WP, being strongly stimulated by HS-L treatment. *ABI5* transcription was not significantly affected by HS-R. These results indicate that exposing leaves to HS stimulated ABA signal transduction, whereas HS-R induced up-regulation of ABA synthesis in the stressed roots, with the synthesised ABA most likely then being transported to leaves.

The observed CK dynamics agree well with these changes (Figures 6, 7, 9). After 6 h under HS-WP conditions, levels of the most physiologically active CK, tZ, were suppressed whereas HS-L caused its concentration to remain at the control level. In the case of HS-WP, this regulation may reflect the impact of HS on roots, which are the primary location of CK production; down-regulation of CK biosynthesis might subsequently affect their transport (as indicated by the levels of tZ riboside and phosphate; Supplementary Table 3). This hypothesis is consistent with the strong reduction of tZ levels observed under HS-R conditions, which coincided with down-regulation of the expression of CK type-A response regulators (e.g., *RR4*). HS-WP caused the strongest CK down-regulation, indicating that HS-WP induced the greatest overall stress. The down-regulation of tZ was partially compensated by an increase in cZ, CK closely related with stress responses. This CK has much weaker effects on cell division (e.g., in tobacco, Gajdošová et al., 2011), but may preserve other CK functions important during stress responses—stimulation of antioxidant system (Li et al., 2014), stabilisation of the photosynthetic apparatus (Cortleven et al., 2019), and promotion of protective compound production (Hare et al., 1997). Expression of the CK biosynthetic gene *IPT9* (a homologue of *AtIPT2*) increased mildly under HS-WP conditions, suggesting that it might contribute to cZ synthesis (Miyawaki et al., 2006); *IPT10* (a homologue of *AtIPT9*) might also be involved. Coincidence of elevated cZ content with the suppression of CKX activity after HS-WP and HS-L may indicate the tendency to diminish cZ degradation in order to keep its level sufficiently high. Further decrease of CKX activity after acclimation (in the case of HS-WP) may reflect protective effect of this pre-treatment.

The significant increase in the leaf auxin content under HS-WP and HS-L conditions (Figure 5) is consistent with the reported HS stimulation of the *YUCCA* auxin biosynthetic genes (Feraru et al., 2019). IAA plays an important role in HS-induced thermomorphogenesis, especially leaf hyponasty as well as stem/hypocotyl elongation (Küpers et al., 2020; Li et al., 2021). Moreover, auxin signalling is promoted by negative effect of HS on the levels of PILS6 protein, auxin carrier implicated in intracellular auxin distribution, which limits nuclear auxin availability (Feraru et al., 2019). Sheldrake (2021) hypothesised that enhanced IAA biosynthesis may be caused by stress-induced protein degradation, which would increase levels of the IAA precursor tryptophan.

Significant increase of JA as well as of its active conjugate JA-Ile in HS treated leaves demonstrates its important role in the HS response (Figure 3). Positive effect of acclimation on JA levels in the case of HS-WP indicated JA protective function. These data are in accordance with Clarke et al. (2009), who found elevation of JA and JA-Ile upon HS in *Arabidopsis thaliana*. They reported important role of JA and SA in basal thermotolerance. In rice, however, moderate SA decrease was detected upon HS-WP in leaves (irrespective of acclimation; Figure 4). The levels of benzoic acid were significantly elevated in all organs of HS-R treated plants (regardless acclimation; Supplementary Table 3). The lack of correlation between benzoic acid and SA levels might be caused by the fact that benzoic acid could be metabolised also to other compounds, like e.g., vanillic acid (Wang et al., 2019), or stored into vacuoles, which may be related to the stress memory (Widhalm and Dudareva, 2015). In contrast to SA, HS-WP treatment significantly increased the level of ACC, direct precursor of ethylene. ACC content in leaves seems to correlate with the stress strength, as acclimation as well as stress targeting were associated with ACC decrease almost to control level.

The stress responses of leaves were relatively weak (Figure 9); the expression of the key HS induced transcription factor, *HSFA2d*, was not significantly affected by any HS treatment. This may be due to its dynamics. The *HSFA2d* expression probably peaked within the 6-h HS treatment period, before measurements were performed. This agrees with the study of Cheng et al. (2015), who reported maximum of *HSFA2d* expression in rice only 15 min after the onset of the HS. The *HSFA2d* response observed in leaves was weaker than that seen in crowns, which may be related to the stress management strategy of rice—suppression of leaf growth caused by severe stress is not reverted after stress release; instead, a new leaf quickly emerges from the crown.

The expression of the high-molecular weight HSPs *HSP90.2* and *HSP90.3* was moderately increased by HS-WP, although this increase was considerably weaker in acclimated plants, indicating that acclimation enhanced stress tolerance (Figure 9). No stimulation in their expression was observed after HS-L, while HS-R significantly increased the expression of *HSP90.2* in leaves (like that of *SIG5*). Acclimation strengthened this effect in the non-targeted organs, promoting stress readiness. Conversely, the low-molecular weight *HSP26.2* expression was up-regulated more strongly after HS-WP in leaves than in the other organs, independently of acclimation. Under HS-L conditions, its expression increased moderately, not changing significantly under HS-R. It seems that its expression response is characteristic for leaves, and reacts readily to leaf-targeted stress. Small HSPs are generally up-regulated by different stresses; they bind to aggregating proteins and enable their refolding by high molecular weight HSPs, which serve as chaperones (Sun et al., 2002).

Many stresses, including HS, increase ROS production. However, the main sources of ROS are photosynthesis and respiration (Mittler, 2002). The activity of CAT and APX did not change significantly in leaves in our experiments (except CAT decrease after HS-L; Figures 10–12). Contrarily, SOD activity increased under HS-R with prior acclimation. Comparison of *SOD* expression profiles (Figure 9) revealed that the mitochondrial *MnSOD* (*MSD*) showed increasing tendency by all HS treatments. The central role of mitochondria in HS responses is reflected by the stimulation of *AOX* expression. This mitochondrial enzyme reduces electron flow *via* the cytochrome c respiration pathway by maintaining the oxidised state of the upstream electron-transport components, thereby reducing oxidative stress (Maxwell et al., 1999). HS-WP increased the expression of *AOX1a*, *AOX1c*, and especially *AOX1b*, which may indicate strong oxidative stress. Conversely, the expression of these enzymes was only mildly increased by the targeted HS treatments.

Pronounced effect of the HS-WP and HS-L treatments on leaf responses was showed by PCA (Figure 8); together with substantial difference between HS-R and shoot-targeted HS responses.

### Crown Stress Responses

This meristematic organ is preferentially protected during stress, which is consistent with the “optimal defence strategy” proposed by McKey (1974) for coping with herbivore attack. This hypothesis is based on the assumption that when plants under severe stress cannot efficiently protect all their organs, they need preferentially protect the tissues “most valuable” for their fitness and survival. For example, preferential protection of the apical meristem enabled tolerance of severe salt stress in the halophyte *Thellungiella halophila* (Prerostova et al., 2017). In accordance with this concept, rice crowns maintained the highest expression of *HSFA2d, HSP26.2*, and *SIG5* after HS-WP, and of *HSP90.3* after HS-WP with prior acclimation (Figure 9).

Direct HS-WP and HS-L treatments also increased APX activity (Figure 12) in crowns, which is in accordance with the elevated APX activity observed in rice seedlings subjected to drought (Sharma and Dubey, 2005; Pandey et al., 2014) or salt stress (Tsai et al., 2004). In the case of HS-R, direct HS-R strongly promoted SOD activity (Figures 10, 12). The expression of mitochondrial *MnSOD* and *AOX* genes was enhanced by HS, especially with prior acclimation, suggesting efficient protection of the mitochondria against oxidative stress. Plastid *FeSOD* expression also increased in acclimated plants, indicating that acclimation enhanced stability of the photosynthetic apparatus (Figures 1, 9).

HS-WP significantly increased JA-Ile, ACC and IAA levels in crowns while reducing tZ levels by biosynthesis inhibition (Figures 5, 6, Supplementary Figure 2 and Supplementary Table 3). Elevation of JA-Ile, in contrast to unaffected levels of JA, may indicate strengthening of JA signal transduction in preferentially protected crowns, which stimulates expression of transcription factors of the WRKY superfamily (Li et al., 2010, 2011). Increased ACC content is in accordance with protective ethylene function (Poór et al., 2021). Elevated IAA levels might be a consequence of diminished auxin polar transport (Bielach et al., 2017). Strong down-regulation of tZ may be associated with HS imposed growth arrest. Low tZ levels (in combination with non-affected cZ content) diminished necessity of CK degradation by CKX (Figure 13). PCA (Figure 8) revealed that the hormone profiles of crowns under HS-WP conditions partially overlapped with that of control plants, which may indicate the efficient protection of these organs.

Targeted HS treatments efficiently stimulated defence mechanisms in crowns. HS-L treatment was characterised by increased levels of ABA and especially cZ (to a much greater extent than in the other organs), and APX activity, independently of acclimation (Figures 2, 7, 12). cZ levels seem to be promoted by its transport, especially from roots (Supplementary Table 3). Unaffected tZ synthesis may indicate preservation of the sink strength (Figure 6 and Supplementary Table 3). HS-R caused an even stronger up-regulation of ABA levels, and it elevated also the other stress-related hormones JA and JA-Ile as well as benzoic acid and the expression of *AOX* isoenzymes (Figures 2, 3, 9, Supplementary Figure 2, and Supplementary Table 3). Levels of SA and IAA increased as well, but this change was non-significant due to the high variation among the samples (Figures 4, 5).

Acclimation had no significant effect in the HS-L case (see PCA analysis; Figure 8) but the HS-R response differed strongly between acclimated and non-acclimated plants. The acclimated HS-R plants had similar hormonal profile as control, indicating the positive protective role of the acclimation. The elevated activity of APX in acclimated plants under HS-R conditions suggests that acclimation accelerated the stress responses in crowns (Figure 12). The increase in SOD activity after direct HS-R was greater than that induced by HS-L, suggesting that HS targeted to roots causes greater ROS production in crowns than HS applied to leaves (Figure 10).

HS-WP as well as HS-L treatment with prior acclimation reduced CAT activity in crowns (Figure 11). This is consistent with the results of Sharma and Dubey (2005) and Pandey et al. (2014), who found that drought reduced CAT activity in rice. However, no significant effect was detected in salt-stressed rice shoots and roots (Tsai et al., 2004). It should be noted that rice contains 3 catalase isoenzymes, each of which may be regulated differently. For example, HS increased *CAT1* and *CAT3* expression in tobacco while reducing that of *CAT2* (Lubovská et al., 2014).

### Root Stress Responses

In roots, like in crowns, HS-WP promoted strong stimulation of expression of *HSFA2d*, *HSPs* (especially after acclimation), and *SIG5* (Figure 9). Similar up-regulation of these genes was also observed after HS-R following acclimation. These changes were more extensive than in crowns and leaves. The data are consistent with conclusions of Bellstaedt et al. (2019), who found that roots can sense and respond to temperature in a shoot- and light-independent manner, whereas shoot temperature responses require both local and systemic processes.

Direct HS-WP treatment also suppressed SOD and CAT activity while increasing APX one and SA levels (Figures 4, 10–12). The data seem to indicate organ-specific SA functions. SA was reported to promote antioxidant system as well as synthesis of defence-related proteins (Wang and Li, 2006; Wang et al., 2010). Exogenous SA reduced HS-induced membrane damage (Wassie et al., 2020). HS applied to roots (HS-WP and HS-R) strongly reduced the content of JA and especially of JA-Ile, HS-R also diminished ACC (Figure 3, Supplementary Figure 2, and Supplementary Table 3). Contrarily, HS-L treatment increased the levels of these hormones, together with elevation (even if statistically insignificant) of ABA. The elevated contents of ABA metabolites indicates active ABA turn-over (Figure 2 and Supplementary Table 3). The distinct responses of these phytohormones to HS-L treatment in roots were clearly demonstrated by PCA (Figure 8). Under HS-L conditions, acclimation did not affect significantly hormonal levels. However, it affected antioxidant enzyme activities (Figures 10–12), reducing the activity of both CAT and APX. HS-L treatment thus efficiently promoted defence responses in non-exposed organs. These results demonstrate rapid inter-organ communication in response to targeted stress.

HS-R treatment substantially reduced levels of IAA in roots, independently of acclimation. This may be related to the suppression of auxin polar transport under stress conditions (Bielach et al., 2017), which corresponds well to the low levels of IAA conjugates under direct HS-WP and HS-R treatments (Supplementary Table 3). This is in agreement with the cold stress induced inhibition of basipetal auxin transport (Shibasaki et al., 2009), while salinity affected the balance between endocytosis and exocytosis of PIN transporters (Zwiewka et al., 2015). In addition to the reduction in IAA levels, auxin signal transduction in roots may be suppressed by the down-regulation of JA and ethylene (Xu et al., 2020).

Acclimation after HS-WP and HS-R promoted expression of antioxidant enzymes (*AOX*, especially *AOX1b*, and *FeSOD*) and counteracted the stress-induced reduction in JA, ACC and cZ levels (Figures 3, 7, 9 and Supplementary Table 3). Much higher CKX activity in roots in comparison with aboveground tissues (moderately promoted by HS-R and HS-WP after acclimation) reflects the fact that these organs are the main location of CK biosynthesis (Figure 13). The similarity of the responses to the HS-WP and HS-R treatments suggest that acclimation strongly affects the stress response in roots. Under HS-R conditions, acclimation also had a profound positive effect on the expression of ABA biosynthetic genes (*NCED3, NCED5*) and especially on the signalling-related gene *ABI5.* Simultaneously, ABA deactivation was low. This suggests, together with high ABA levels in crowns and leaves, that ABA was actively transported from roots to shoots. No additional effect of acclimation was observed for cytoplasmic *CuZnSOD*, which was up-regulated by targeted HS treatments.

## Conclusion

Prior acclimation greatly strengthened defence responses, especially when roots were subjected to HS (HS-WP, HS-R), suggesting that roots play important roles in HS responses. The lesser protection of leaves may be related to the fact that highly stressed leaves initiate senescence and are eventually replaced by new leaves emerging from crowns. Applying HS targeted to individual organs induced substantial changes in stress-related hormone levels and antioxidant enzyme activity in organs not directly exposed to the stress, which appears to enhance stress tolerance. The main effect of HS-L treatment was to increase levels of JA and JA-Ile in roots, and of cZ in crowns, while also stimulating CAT and APX activity. Conversely, HS-R treatment increased levels of ABA and IAA in leaves and crowns, cZ in leaves, and JA/JA-Ile in crowns, while reducing levels of tZ. Finally, acclimation strongly promoted up-regulation of *HSP90.2* and *SIG5*, which protect the photosynthetic apparatus.

## Data Availability Statement

The original contributions presented in the study are included in the article/Supplementary Material, further inquiries can be directed to the corresponding author/s.

## Author Contributions

RV: conceptualisation and writing—original draft preparation. JJ, RF, LH, and VM: formal analysis. SP, RV and JK: funding acquisition. SP, JJ, PD, RF, LH, VM, and VK: investigation. VK, RV, and SP: methodology. RV, PD, and JK: resources. RV and SP: supervision. AG, JJ, VK, and SP: visualisation. RV, SP, JJ, PD, VM, and JK: writing—review and editing. All authors contributed to the article and approved the submitted version.

## Conflict of Interest

The authors declare that the research was conducted in the absence of any commercial or financial relationships that could be construed as a potential conflict of interest.

## Publisher’s Note

All claims expressed in this article are solely those of the authors and do not necessarily represent those of their affiliated organizations, or those of the publisher, the editors and the reviewers. Any product that may be evaluated in this article, or claim that may be made by its manufacturer, is not guaranteed or endorsed by the publisher.

## Supplementary Material

Supplementary Figure 1 Setup of the insulated vessels.

Supplementary Figure 2 Jasmonoyl-isoleucine.

Supplementary Table 1 List of primers.

Supplementary Table 2 RT-qPCR.

Supplementary Table 3 Phytohormone metabolites.

## Abbreviations

A: acclimation
ABA: abscisic acid
AOX: alternative oxidase
APX: ascorbate peroxidase
CAT: catalase
CK: cytokinin
CKX: cytokinin oxidase/dehydrogenase
cZ: *cis-*zeatin
F_*v*_/F_*m*_: maximum photosystem II quantum yield
HS: heat stress
HS-L: heat stress targeted to leaves
HS-R: heat stress targeted to roots
HS-WP: heat stress targeted to the whole plant
HSF: heat shock factor
HSP: heat shock protein
IAA: indole-3-acetic acid
IPT: isopentenyltransferase
JA: jasmonic acid
JA-Ile: jasmonoyl-isoleucine
NCED: 9-*cis-*epoxycarotenoid dioxygenase
NPQ__*Lss*_: steady-state non-photochemical quenching
PSII: photosystem II
QY__*Lss*_: steady-state photosystem II quantum yield
ROS: reactive oxygen species
RR: cytokinin response regulator
SA: salicylic acid
SIG5: sigma factor 5
SOD: superoxide dismutase
tZ: *trans-*zeatin.
