# Supplementary figures and images for: Heat Stress Targeting Individual Organs Reveals the Central Role of Roots and Crowns in Rice Stress Responses
Source: Front Plant Sci. 2022 Jan 17;12:799249. doi: 10.3389/fpls.2021.799249 (PMC8801461; doi:10.3389/fpls.2021.799249)

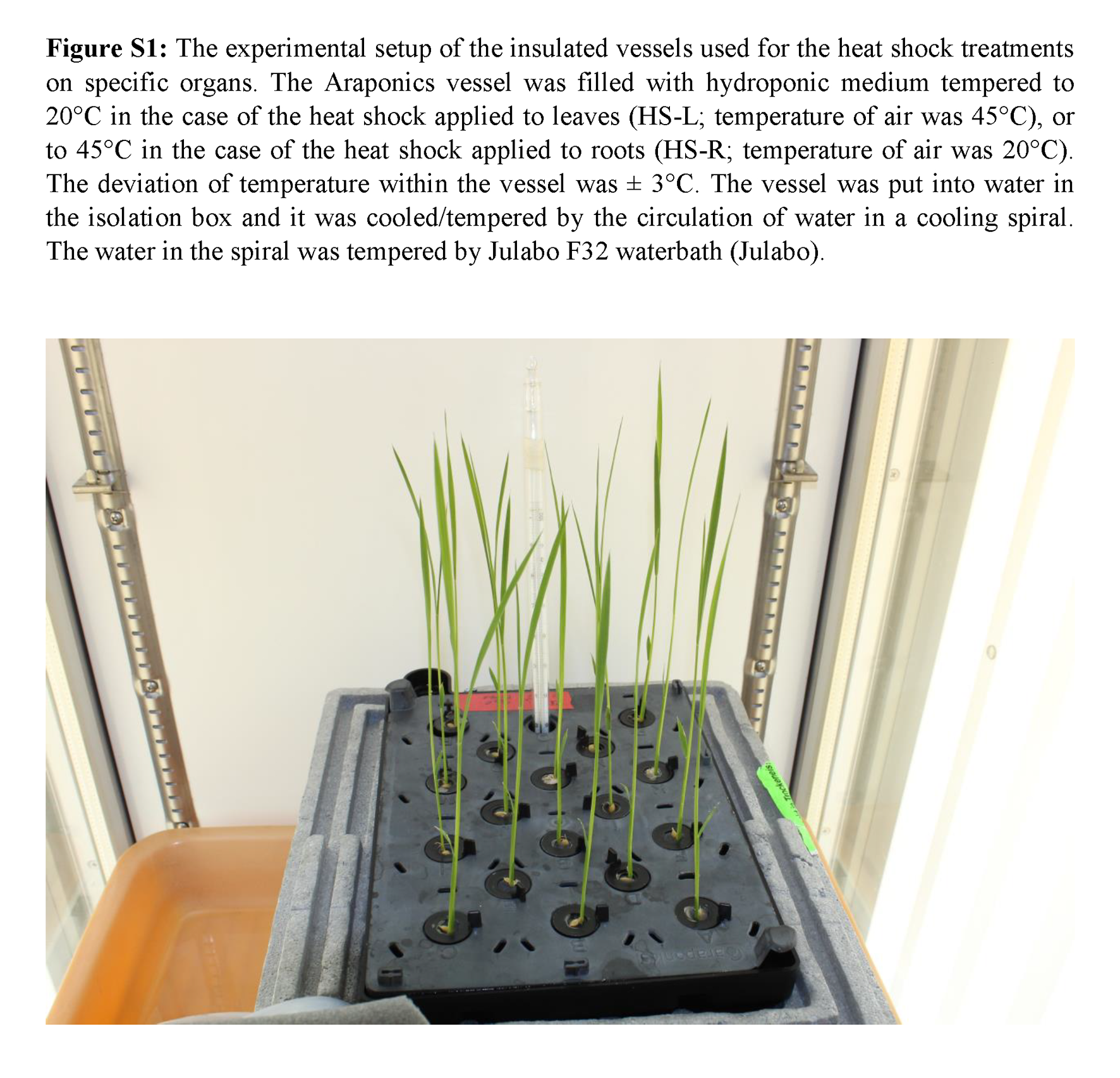

Supplement: Supplementary Figure 1 — Setup of the insulated vessels. [file Image_1.tif]

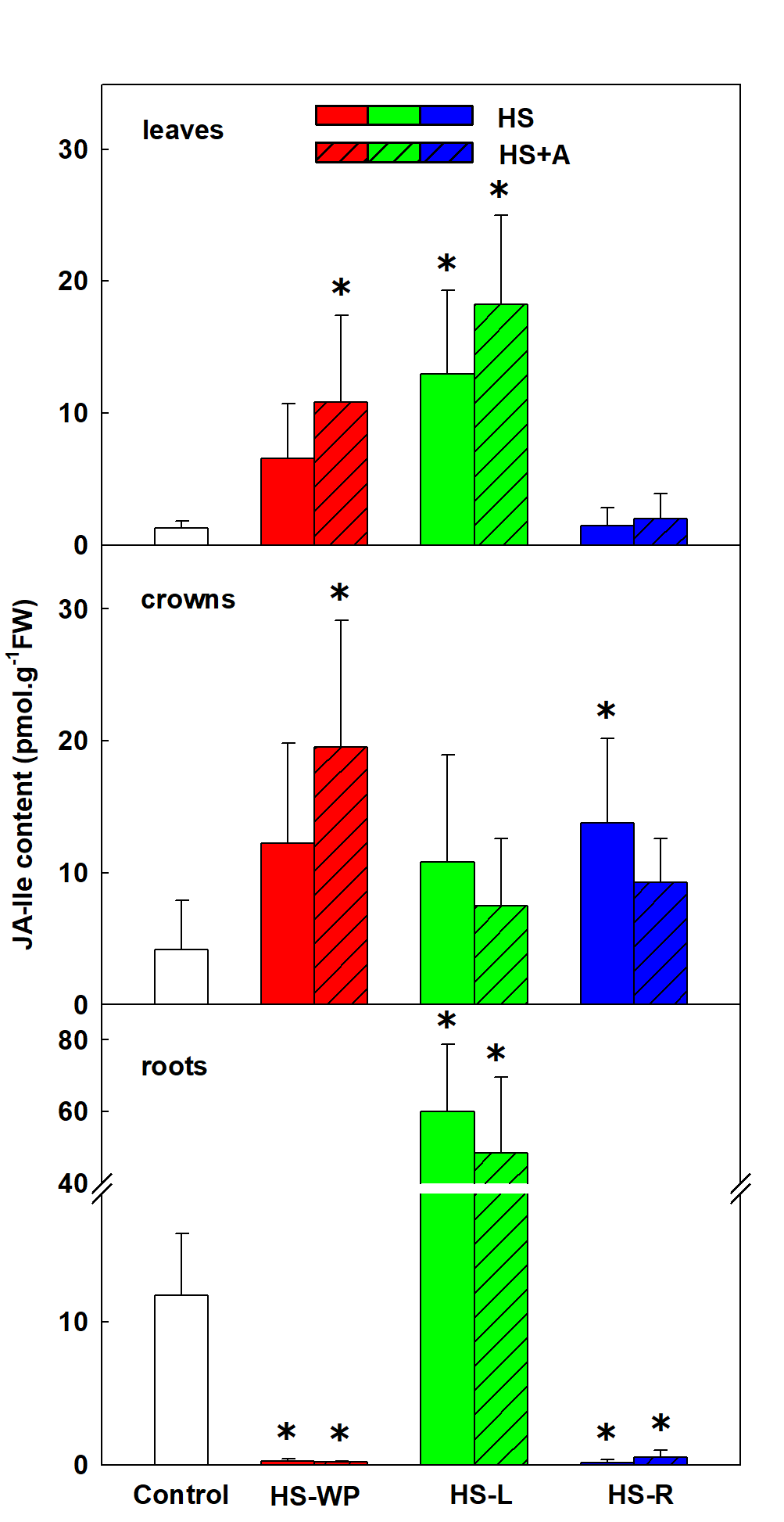

Supplement: Supplementary Figure 2 — Jasmonoyl-isoleucine. [file Image_2.tif]
